# Nodules-associated *Klebsiella oxytoca* complex: genomic insights into plant growth promotion and health risk assessment

**DOI:** 10.1186/s12866-025-04002-7

**Published:** 2025-05-15

**Authors:** Sameh H. Youseif, Fayrouz H. Abd El-Megeed, May S. Soliman, Amr Ageez, Akram H. Mohamed, Saher A. Ali, Amani A. El-Kholy

**Affiliations:** 1 School of Biotechnology, Nile University, Giza, 12677 Egypt; 2 Department of Microbial Genetic Resources, National Gene Bank (NGB), Agricultural Research Center (ARC), Giza, 12619 Egypt; 3 Department of Clinical and Chemical Pathology, Faculty of Medicine, Cairo University, Cairo, Egypt; 4 Faculty of Biotechnology, October University for Modern Sciences and Arts (MSA), 6th October, Giza, 12451 Egypt

**Keywords:** Nodules-associated bacteria, *Klebsiella oxytoca *complex, Genomics, Plant growth promotion mechanisms, Antibiotic resistance genes, Health risk assessment, One health approach

## Abstract

The swift emergence of antibiotic resistance genes (ARGs) across interconnected One Health compartments poses a significant global threat. Although plant growth-promoting (PGP) bacteria possess numerous attributes beneficial to host plants, many of these bacteria also harbor ARGs, necessitating a focused assessment of their negative implications. In this context, here we performed whole genome sequencing of 14 PGP endophytic strains isolated from root nodules of faba beans, belonging to three *Klebsiella oxytoca* species complex (KoSC): *K. grimontii* (*n* = 5), *K. michiganensis* (*n* = 5), and *K. pasteurii* (*n* = 4). We performed comparative genomics, molecular typing, and pangenome analyses on these strains. We identified significant diversity within the KoSC population, classifying the strains into five sequence types (STs), three of which are novel to this study (ST-542, ST-569, and ST-629). Phylogenomic analysis revealed that the bacterial strains clustered more closely by ST than by their source of isolation. Annotation of gene clusters indicated that all assembled genomes are enriched with genes involved in PGP activities, alongside a robust array of genes conferring tolerance to abiotic stresses. Importantly, our findings disclosed that the 14 assembled genomes harbored multiple ARGs, conferring resistance to various antibiotic classes, with 71% of the population classified as multidrug-resistant based on the in vitro antibiotic susceptibility assay. Furthermore, all genomes contained an array of virulence factors critical for survival, pathogenesis, biofilm formation, and root colonization. In conclusion, this study substantiates the hypothesis that certain PGP bacteria may serve as potential reservoirs of multidrug resistance, posing significant public health risks. Thus, the future advancement of bacteria-based biofertilizers should integrate environmental considerations and monitor their impact on antibiotic resistance dissemination in soil ecosystems.

**Supplementary Information:**

The online version contains supplementary material available at 10.1186/s12866-025-04002-7.

## Introduction

The *Klebsiella* genus is an encapsulated, non-motile, rod-shaped member of the *Enterobacteriaceae* family. At the time of writing, the genus *Klebsiella* contained 24 species of bacteria with validly published names and three species with a non-valid name (www.lpsn.dsmz.de). Among all species, *Klebsiella pneumoniae* and *Klebsiella oxytoca* have garnered significant clinical attention as they are responsible for most infectious diseases and play a significant role in the global dissemination of antimicrobial resistance genes (ARGs) [1, 2]. The remarkable genome plasticity of *Klebsiella* [3] has facilitated rapid evolution over the past decade, resulting in the emergence of isolates exhibiting multidrug resistance and hypervirulence (MDR-hv) phenotypes [4]. MDR-hv *Klebsiella* isolates have expanded their resistance profile beyond commonly used antibiotics to include last-line treatments such as carbapenems, tigecycline, and colistin [4]. Alarmingly, studies indicate exceptionally high levels of antibiotic resistance among *Klebsiella* strains in various African countries, with resistance rates soaring to 97.17% in certain regions [5]. This situation underscores the urgent need for effective monitoring and intervention strategies to address the growing threat of *Klebsiella* infections.

In recent years, the use of whole-genome sequencing (WGS) in taxonomical studies has led to a greater understanding of the genetic diversity exhibited by *Klebsiella* spp. [6]. It is now understood that the *Klebsiella oxytoca* species complex (KoSC) is a heterogeneous group, comprising multiple species [7]. The chromosomally encoded *β*-*lactamase* gene (*bla*_*OXY*_) showed sequence variability that led to the establishment of nine phylogroups (Ko), corresponding to six valid species and three unnamed new species (taxons one, two, and three) [4]. *K. michiganensis* (Ko1, Ko5), *K. oxytoca* (Ko2), *K. spallanzanii* (Ko3), *K. pasteurii* (Ko4), *K. grimontii* (Ko6), and *K. huaxiensis* (Ko8). It is very challenging to reliably distinguish members of the complex using phenotypic characteristics or standard typing methods [8]. Consequently, several reports indicated that public sequence databases include many genomes that are misclassified as *K. oxytoca* instead of *K. michiganensis* or *K. grimontii* [7, 8]*.*

Strains belonging to the KoSC are versatile pathogens that have been linked to nosocomial infections in humans [2], but they have also been isolated from different environmental reservoirs, including soil [9], plants [10], water, and sewage sludge [7]. Recently, members of the KoSC have attracted considerable attention as a common plant-growth-promoting (PGP) bacteria that can effectively fix atmospheric nitrogen, produce phytohormones, solubilize inorganic phosphate, and induce plant systemic resistance [9, 10]. PGP bacteria possess multiple traits that can be beneficial for their host plants [11]. They contribute significantly to sustainable agriculture by reducing the application of chemical synthetic inputs, improving soil fertility, promoting plant health, and increasing crop productivity [12]. While most PGP bacteria are rhizospheric inhabitants, some are endophytes, which can colonize root tissues and migrate to different plant tissues. Endophytic PGP bacteria, due to their direct contact with plant tissues, are considered unique plant partners and have gained a significant deal of interest in the past decade [13].

Despite the agronomic importance of PGP bacteria, many of them harbor ARGs that can spread to indigenous soil microbial communities through vertical and/or horizontal transfer [14, 15]. This, in turn, increases their transmission to other environmental reservoirs, such as water effluents, animals, and humans [16]. Consequently, crop inoculation with PGP bacteria harboring ARGs may exacerbate the dissemination of antibiotic resistance, thereby intensifying negative effects on ecosystems and public health [15]. Therefore, further investigation into the extent of antibiotic resistance in PGBP is necessary prior to their large-scale application in soils. This is to reduce the risk of resistance transmission to the soil microbiome and eventually their negative effects on human health.

Despite a significant amount of comparative genomic research on KoSC members, most studies have focused primarily on clinical strains associated with infectious diseases. While there are a few reports on the genomic studies of KoSC members with PGP potential [10, 17], genomic analyses of KoSC strains recovered from legume nodules are virtually unavailable due to the limited number of strains isolated from these nodules. This research gap hampers our understanding of their vital role in agriculture and the potential health risks associated with their environmental applications. In a previous study, we isolated 34 endophytic bacteria from root nodules of faba bean that had positive PGP attributes such as siderophore production, ammonia production, and phosphate solubilization [18]. These bacteria significantly enhanced symbiotic nitrogen fixation and improved various growth parameters of faba bean plants under controlled greenhouse conditions [18]. Based on multilocus sequence analysis (MLSA) of three housekeeping genes, we classified 14 strains of the 34 bacteria into the three KoSC: (*n* = 5) *K. michiganensis*; (*n* = 4) *K. pasteurii;* and (*n* = 5) *K. grimontii* [18].

In the current investigation, we employed whole-genome sequencing and comparative analysis to elucidate the molecular mechanisms regulating multiple PGP characteristics exhibited by these bacteria, and we compared our findings with previously reported PGP strains. We provided a detailed taxonomical description of these strains and compared them with those of other *Klebsiella* species. We also hypothesized that these PGP endophytic bacteria might have negative consequences for public health due to antibiotic resistance. To investigate this hypothesis, we identified gene clusters that control antibiotic resistance and virulence factors (if any) and compared them to the genomes of other *Klebsiella* strains found in clinical settings. The obtained results will assist in the identification of bacterial candidates that possess the largest potential for promoting plant growth while exhibiting the least risk for virulence and antibiotic resistance.

## Methods

### Bacterial strains

Fourteen PGP endophytic strains classified into three KoSC were previously isolated from root nodules of faba bean plants [18]. Bacterial identification was performed based on MLSA of three housekeeping genes [18]. The bacterial strains were stored at -80 °C and were deposited in the culture collection of the National Gene Bank (NGB), Agricultural Research Center (ARC), with the following NGB accession codes: NGB-FR1, FR3, FR19, FR21, FR40, FR50, FR52, FR67, FR89, FR100, FR108, FR111, and FR129.

### Antimicrobial susceptibility testing

The antibiotic susceptibility of the 14 strains belonging to KoSC was assayed on Muller-Hinton agar (Oxoid, UK) using the disc diffusion method as described in [19]. The antibiotics (Bioanalyse®, Turkey) used were amikacin 30 µg, gentamicin 10 µg, amoxicillin/clavulanic acid 20/10 µg, aztreonam 10 µg, chloramphenicol 30 µg, piperacillin/tazobactam 100/10 µg, meropenem 10 µg, ceftazidime 30 µg, levofloxacin 5 µg, ciprofloxacin 5 µg, trimethoprim/sulfamethoxazole 1.25/23.75 µg, and ampicillin 10 µg. The zone of growth inhibition surrounding the disc was measured in millimeters to evaluate the antibiotic inhibitory effect. The results obtained were interpreted according to the Clinical and Laboratory Standards Institute (CLSI) guidelines [20].

### DNA extraction and WGS

Bacterial DNA was isolated from fresh subcultures using the GeneJET genomic DNA purification kit (Thermo Fisher Scientific, USA) according to the manufacturer’s instructions. The quality of the extracted DNA was confirmed using DeNovix DS-11 (DeNovix, USA). Genomic DNA was stored at -20 °C. WGS was performed at the Faculty of Medicine, Cairo University, Egypt, using Illumina technology. Paired-end short-read sequencing libraries were constructed using the Nextera XT DNA library preparation kit (Illumina, USA). Sequencing was carried out with the MiSeq reagent kit 600 v3 (Illumina, USA) on the Illumina MiSeq instrument to produce 301 base pair paired-end reads.

### Genome assembly and annotation

The quality of raw reads was checked using FastQC v0.12.1 (http://www.bioinformatics.babraham.ac.uk/projects/fastqc/). Low-quality reads, Illumina adapters, and bases with quality lower than Q30 were filtered using Trimmomatic v0.39 (http://www.usadellab.org/cms/?page=trimmomatic). The resulting reads were assembled de novo using Unicycler v.0.4.8 assembly pipeline (https://github.com/rrwick/Unicycler) to identify strain-specific variations and discover novel sequences that may be missed by aligning to a reference genome. This approach also provides an unbiased genome representation and enables independent annotation, revealing new genes and pathways specific to the sequenced genome [21, 22]. The completeness and contamination of the assembled genomes were checked using CheckM v1.1.6 [23]. Genome annotation was performed using the Prokaryotic Genome Annotation Pipeline (PGAP; v6.0) available at NCBI [24], and the genome annotation tool in the Bacterial and Viral Bioinformatics Resource Center (BV-BRC) v3.39.3 webtool (https://www.bv-brc.org/). The BV-BRC uses the RAST tool kit [25] to accurately predict genes and protein functions, metabolic pathways, and subsystems. The gene functions were investigated using the Kyoto Encyclopedia of Genes Genomes (KEGG) database (https://www.kegg.jp/) via the BlastKOALA v3.0 tool.

### Taxonomy, phylogenomic analysis, and sequence typing (ST) of *Klebsiella* strains

The genomic-based taxonomical identification of the 14 strains belonging to KoSC sequenced here was done using the Type (Strain) Genome Server (TYGS) (https://tygs.dsmz.de/) with the Genome BLAST Distance Phylogeny method (GBDP) following the default parameters [26]. The species delineation in the TYGS database is based on Digital DNA:DNA hybridization (dDDH) values calculated using the recommended settings of the GGDC v3.0 (https://ggdc.dsmz.de).

Additionally, the genetic relatedness of the 14 strains sequenced here to 151 publicly available genomes from the KoSC was analyzed using the Bacterial Genome Tree tool (parameter 500 genes) available at the BV-BRC webtool (https://www.bv-brc.org/) [27]. The 151 genomes were selected based on their source of isolation and assembly level. The phylogenomic analysis also included five genomes, representing the type strains of the following *Klebsiella* species: *K. africana, K. indica, K. pneumoniae, K. quasipneumoniae,* and *K. variicola.* The genomic features of the comparing strains were retrieved from BV-BRC and GenBank servers. The list of complete genomes is shown in Supplementary Table S1. The statistics of tree analysis and gene families generated by the BV-BRC webtool are shown in Supplementary Tables S2 & S3, respectively.

Sequence types (STs) for all *Klebsiella* genomes were determined using the Public Databases For Molecular Typing and Microbial Genome Diversity (PubMLST) with the *K. oxytoca* scheme (https://pubmlst.org/organisms/klebsiella-oxytoca). The resulting Newick-format tree was visualized and annotated using the Tree Visualization By One Table (tvBOT) webtool v2.121 (https://www.chiplot.online/tvbot.html) [28].

To precisely determine species identity within the KoSC, the average nucleotide identity (ANI) among the 14 *Klebsiella* strains studied here and type strains from closely related *Klebsiella* species was computed using IPGA v1.09 (https://nmdc.cn/ipga/) [29]. Comparative genomic circle mapping of each genospecies (*K. michiganensis, K. grimontii,* and *K. pasteurii*), containing our strains and the phylogenetically closest type strain, was generated using BLAST Ring Image Generator (BRIG v0.95-dist) software [30].

### Pan-genome analysis

Pan-genome analysis was performed using the IPGA pipeline (https://nmdc.cn/ipga/) [29] with default settings (a minimum sequence identity threshold of 70%). This analysis aimed to identify core genes (shared genes among all strains), accessory genes (dispensable genes present in some strains), and strain-specific genes (genes exclusive to one strain).

### Genome mining of PGP and abiotic stress tolerance genes

We screened the annotated genes associated with PGP mechanisms (N_2_-fixation, P-solubilization, indole acetic acid (IAA) biosynthesis, and iron acquisition), as well as those related to abiotic stress tolerance, in the 14 genomes sequenced in this study. Additionally, we compared these findings with the genomes of seven previously published bacterial strains known to possess PGP properties. These bacterial strains include *K. grimontii* Kd70 (GenBank: LGRU00000000.1), *K. variicola* 342 (GenBank: CP000964.1), *K. variicola* GN02 (GenBank: CP031061.1), *K. variicola* UC4115 (GenBank: DAMLKG000000000.1), *K*. *variicola* Sck8 (GenBank: GCA_002810535.1), *Bacillus velezensis* K1 (GenBank: JAADAB000000000.1), and *Saccharibacillus brassicae* ATSA2 (GenBank: CP041217.1). The heat maps were generated based on the presence and absence of PGP genes in the respective strains using the TBtools-II software v0.665 [31]. We evaluated the activity of genes encoding abiotic stress tolerance by screening the growth of 14 *Klebsiella* strains studied here in vitro under wide ranges of salt concentrations and high temperatures as previously described in [32]. The salt tolerance of bacteria was assessed by inoculating 10 µl of overnight culture (approximately 1 × 10⁸ cells ml⁻^1^) onto Yeast Mannitol Agar (YMA) plates with 0.5%, 1%, 2%, 3.5%, 4%, 4.5%, and 5% (w/v) NaCl. Temperature tolerance was tested by incubating at 30, 37, 40, 42, 45, and 50 °C. After 24 h, plates were examined for bacterial growth.

### Virulome analysis, ARG predictions, and mobile genetic elements (MEGs) detection

The antibiotic resistance profile of the 14 genomes in this study was carried out using Resfinder v4.6 (https://genepi.food.dtu.dk/resfinder) at the Center for Genomic Epidemiology (CGE) database with 90% nucleotide identity. Then, the resistome analysis and predictions were done using the online RGI Resistance Gene Identifier v6.0.3 available at The Comprehensive Antibiotic Resistance Database (CARD; https://card.mcmaster.ca). The phenotypic resistance of the 14 strains was compared to the genetic predictions of antimicrobial resistance derived from their assembled genomes. The concordance rate was determined as the percentage of strains exhibiting alignment between phenotypic resistance and the presence of corresponding resistance determinants in their genomes, relative to the total number of isolates. The heatmap demonstrating concordance and discordance was generated using the ggplot R package (R v4.3.2).

The virulence factors were identified using the online VF analyzer platform available at the virulence factor database (VFDB, http://www.mgc.ac.cn/VFs/). We compared the antibiotic resistance and virulome profiles of the 14 studied genomes to those of three type strains: *K. grimontii* 06D02^T^ (GenBank: FZTC00000000.1), *K*. *michiganensis* DSM25444^T^ (GenBank: PRDB00000000.1), and *K. pasteurii* SB6412^T^ (GenBank: CABGHC000000000). We also include six reference strains from the KoSC: *K. grimontii* 2,481,359 (GenBank: CP067380.1), KD70 (GenBank: NZ_LGRU00000000.1), MBTK-1 (GenBank: PDEL01000001.1); *K. michiganensis* 23999A2 (GenBank: JANFNZ000000000.1); and *K. pasteurii* Kox205 (GenBank: CP089403.1), BDA 134–6 (GenBank: CP064784.1). We selected these reference genomes based on their isolation source and assembly level. The heat maps were generated based on the presence and absence of virulent genes and ARGs in the respective strains using the TBtools-II software v0.665 [31].

To identify plasmids and other mobile genetic elements (MGEs) associated with ARGs, the assembled genomes sequenced in this study were analyzed using the PlasmidFinder tool v2.1 and the MobileElementFinder tool v.1.0.3 available at the CGE database (https://www.genomicepidemiology.org/services/) following the default parameters. Besides, the MGEs were further annotated and analyzed using the mobileOG-db database v1.6 [33] available in the Proksee webtool v1.1.3 [34]. The ARGs associated with MGEs in each genome were identified using the CARD webtool; (https://card.mcmaster.ca).

## Results

### In vitro antibiotic susceptibilities

We assessed the antimicrobial susceptibility of 11 antibiotics using the disc diffusion method (Table 1). While the majority of the 14 test strains exhibited sensitivity to most of the tested antibiotics, a significant proportion (71%, 10/14 strains) were classified as MDR, showing non-susceptibility to at least one antimicrobial agent across three or more classes. The strains identified as MDR included *K. michiganensis* (NGB-FR1, FR3, FR19, FR89, FR129), *K. pasteurii* (NGB-FR50, FR52, FR108), and *K. grimontii* (NGB-FR67 and FR111). These strains demonstrated resistance to ampicillin, ceftazidime, and amoxicillin/clavulanic acid, which are categorized within the penicillins, cephems, and β-lactam combination agents, respectively.

**Table 1:** The antibiotic susceptibility of different *Klebsiella* strains sequenced in this study

**Antibiotic class**	**Antibiotic Sub-class**	**Antibiotic**	***K. michiganensis***					***K. pasteurii***				***K. grimontii***				
			**NGB-FR1**	**NGB-FR3**	**NGB-FR19**	**NGB-FR89**	**NGB-FR129**	**NGB-FR49**	**NGB-FR50**	**NGB-FR52**	**NGB-FR108**	**NGB-FR21**	**NGB-FR40**	**NGB-FR67**	**NGB-FR100**	**NGB-FR111**
β-lactam Penicillins	Aminopenicillins	Ampicillin	R (0)	R (0)	R (0)	R (0)	R (0)	R (0)	R (0)	R (0)	R (0)	R (0)	R (0)	R (0)	R (0)	R (0)
β-lactam Cephems	Cephalosporins III	Ceftazidime	R (15)	R (10)	R (16)	R (0)	R (16)	I (20)	R (12)	R (11)	R (10)	S (22)	S (21)	R (10)	S (21)	R (10)
β-lactam Penems	Carbapenems	Meropenem	S (30)	S (39)	S (32)	S (32)	S (30)	S (39)	S (38)	S (34)	S (34)	S (37)	S (39)	S (36)	S (37)	S (33)
β-lactam combination agents	-	Amoxicillin/ Clavulanic acid	R (0)	R (0)	R (12)	R (0)	R (0)	R (0)	R (0)	R (0)	R (0)	R (0)	R (0)	R (0)	R (0)	R (0)
	-	Piperacillin/ Tazobactam	S (25)	S (30)	S (26)	S (27)	S (26)	S (30)	S (27)	S (28)	S (24)	S (26)	S (27)	S (25)	S (28)	S (23)
β-lactam monobactams	-	Aztreonam	S (31)	S (31)	S (29)	S (32)	S (31)	S (34)	S (33)	S (32)	S (35)	S (29)	S (29)	S (29)	S (30)	S (32)
Non-β-Lactam Aminoglycoside	-	Amikacin	S (24)	S (20)	S (24)	S (24)	S (24)	S (20)	S (21)	S (18)	S (19)	S (23)	S (21)	S (21)	S (19)	S (25)
	-	Gentamicin	S (22)	S (22)	S (21)	S (21)	S (24)	S (20)	S (22)	S (21)	S (21)	S (21)	S (23)	S (23)	S (23)	S (22)
Non-β-Lactam Fluoroquinolone	Fluoroquinolones	Ciprofloxacin	S (41)	S (38)	S (39)	S (40)	S (38)	S (39)	S (37)	S (41)	S (39)	S (40)	S (40)	S (40)	S (42)	S (35)
	Fluoroquinolones	Levofloxacin	S (34)	S (36)	S (34)	S (35)	S (33)	S (31)	S (35)	S (37)	S (35)	S (38)	S (39)	S (36)	S (41)	S (34)
Non-β-Lactam Phenicol	-	Chloramphenicol	S (33)	S (31)	S (30)	S (33)	S (32)	S (29)	S (30)	S (30)	S (31)	S (27)	S (27)	S (29)	S (29)	S (27)
Non-β-Lactam Folate pathway antagonists	Combination	Trimethoprim/ Sulfamethoxazole	S (27)	S (31)	S (29)	S (29)	S (28)	S (31)	S (30)	S (27)	S (26)	S (30)	S (28)	S (26)	S (27)	S (33)

NGB codes represent the National Gene Bank accession numbers for the 14 bacterial strains. Data represents the inhibition zone (mm). R (antibiotic-resistant phenotype), S (antibiotic-sensitive phenotype), and I (antibiotic-intermediate phenotype). Antibiotic classes and results are interpreted according to the CLSI [18]

### General genome features

The genomes of 14 strains were sequenced and de novo assembled. Genome sequencing and assembly summary features for the 14 strains are shown in Table 2. The genome size ranged from 5.73 to 6.08 Mb, represented by 26–106 contigs, with L50 values that range from 4 to 11 (average = 7.5) and N50 values that range from 187,285 to 532,684 bp (average = 359,985 bp). The maximum contig length ranges from 507,809 to 1,196,605 bp, and the minimum contig length ranges from 308 to 809 bp. The GC% of the 14 genomes ranged from 55.25 to 55.97%.

**Table 2:** Genomic features of the 14 *Klebsiella* strains sequenced in this study according to the BV-BRC server

**KoSC genome**	**Genome ID**	**Genome Name**	**Genome Statistics**					**Quality Statistics**			**Annotation Statistics**				
			**Contigs**	**Genome size (bp)**	**GC Content (%)**	**Contig L50**	**Contig N50**	**Compl-eteness**	**Contami-nation**	**Hetero-geneity**	**tRNA**	**rRNA**	**CDS**	**Hypoth. CDS**	**PLFAM CDS**
NGB-FR1	1134687.762	*K. michiganensis*	51	5937438	55.97	8	284943	100	0.22	0	75	3	5845	836	5605
NGB-FR3	1134687.758	*K. michiganensis*	48	5944886	55.97	7	333309	100	0.22	0	72	3	5851	838	5610
NGB-FR19	1134687.759	*K. michiganensis*	51	5943594	55.97	8	288040	100	0.22	0	77	3	5852	839	5613
NGB-FR89	1134687.760	*K. michiganensis*	66	6018396	55.86	8	267815	100	0.98	7.14	77	3	5976	898	5706
NGB-FR129	1134687.761	*K. michiganensis*	45	5945363	55.97	7	333259	100	0.22	0	75	3	5840	829	5607
NGB-FR49	2587529.18	*K. pasteurii*	105	6079594	55.25	7	285235	100	0.96	0	77	3	6064	920	5716
NGB-FR50	2587529.17	*K. pasteurii*	99	6079302	55.25	5	347344	100	0.96	0	77	3	6058	915	5715
NGB-FR52	2587529.16	*K. pasteurii*	106	6081593	55.25	7	347318	100	0.96	0	77	3	6075	929	5723
NGB-FR108	2587529.15	*K. pasteurii*	66	5761980	55.54	11	187285	100	0.37	0	71	4	5632	787	5353
NGB-FR21	2058152.249	*K. grimontii*	27	5733048	55.92	4	532702	100	0.43	0	73	4	5560	676	5358
NGB-FR40	2058152.251	*K. grimontii*	28	5733386	55.92	4	427497	100	0.43	0	73	4	5565	681	5359
NGB-FR67	2058152.250	*K. grimontii*	30	5734063	55.92	4	427497	100	0.43	0	73	4	5557	672	5359
NGB-FR100	2058152.248	*K. grimontii*	31	5731571	55.82	5	377939	100	0.43	0	71	3	5563	678	5362
NGB-FR111	2058152.247	*K. grimontii*	26	5733375	55.92	4	532684	100	0.43	0	73	4	5561	676	5355

NGB codes represent the National Gene Bank (NGB) accession numbers for the 14 bacterial strains sequenced in this study

Genome ID according to the BV-BRC database (https://www.bv-brc.org/). PLFams: Proteins with PATRIC genus-specific family

### Phylogenomic analysis of strains belonging to KoSC

**Fig. 1:**
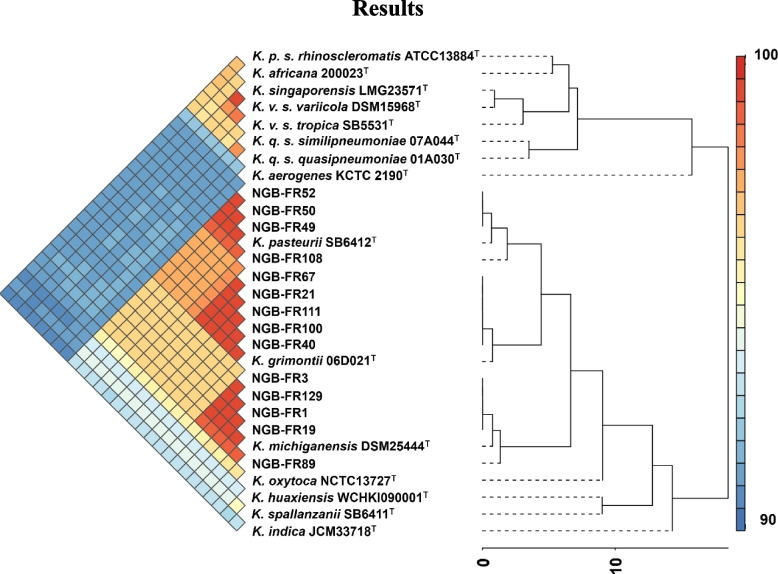
Pairwise comparison of average nucleotide identity (ANI) values between the 14 strains sequenced in this study and closely related *Klebsiella* species (*n* = 15). The heatmap displays ANI values, with the color gradient representing intergenomic similarities ranging from 90 to 100%, where higher values denote greater similarity. The dendrogram illustrates hierarchical clustering, reflecting the evolutionary relationships among strains. The scale below the dendrogram, from 0 to 10, indicates distance based on ANI, with higher values signifying greater dissimilarity

To provide the accurate taxonomic position of the 14 strains studied here, a whole-genome-based taxonomic analysis was performed using dDDH species clustering in the TYGS platform (Supplementary Fig. S1). We also confirmed the reliability of evolutionary distance based on dDDH comparisons by calculating the ANI values among the 14 strains and closely related type strains (Fig. 1 and Supplementary Table S4). The dDDH (d4, species-level cutoff = 70%) and ANI (cutoff = 96%) analyses showed that strains NGB-FR1, 3, 19, 89, and 129 were most closely related to *K. michiganensis* DSM 25444^ T^ (dDDH = 89.7–95.1%; ANI = 98.7–99.3%). Strains NGB-FR21, 40, 67, 100, and 111 were taxonomically assigned to *K. grimontii* 06D021^T^ (dDDH = 95.5%; ANI = 99.2–99.3%). Strains NGB-FR49, 50, 52, and 108 were tightly affiliated to *K. pasteurii* SB6412^T^ (dDDH = 84.6–95.7%; ANI = 98.1–99.3%).

**Fig. 2:**
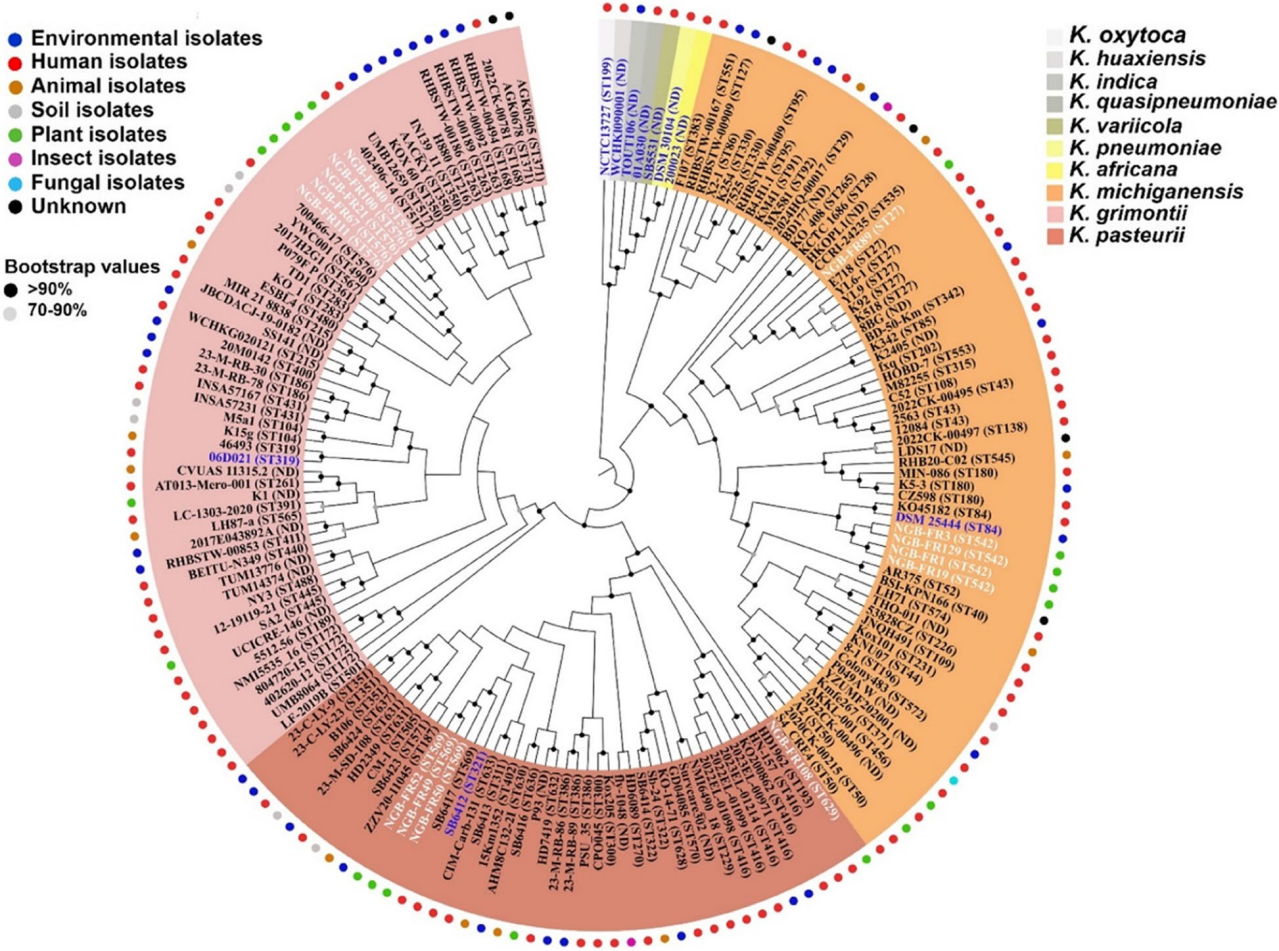
Phylogenomic tree of the strains sequenced here compared to representative *Klebsiella* strains (*n* = 170) constructed with the BV-BRC Bacterial Genome Tree service. Phylogenomic analysis revealed that the bacterial strains clustered more closely by sequence types than by their source of isolation. The tree was generated using the Codon Tree method from 500 single-copy shared genes. Support values were generated using 100 rounds of the “Rapid” bootstrapping option of the RAxML program. Genomes in blue represented type strains, whereas genomes in white represented the 14 strains sequenced in this study. The ring encircling the phylogeny signifies the reported isolation source for each strain

We characterized five sequence types within the 14 strains sequenced in the current study (Fig. 2 and Supplementary Table S1). *K*. *michiganensis* strains: NGB-FR1, 3, 19, and 129 belonged to ST542, whereas NGB-FR89 belonged to ST27. All *K. grimontii* strains: NGB-FR21, 40, 67, 100, and 111 belonged to ST576. *K. pasteurii* strains: NGB-FR 49, 50, and 52 belonged to ST569, but NGB-FR 108 belonged to ST629. This indicates the dominance of ST542, ST576, and ST569 within our collection. Notably, ST542, ST569, and ST629 are reported for the first time in this study.

To further elucidate the phylogenomic relationships between the 14 strains sequenced in this study and those previously deposited in GenBank, we analyzed the genome sequences of 151 strains belonging to the KoSC alongside five type strains from other *Klebsiella* species (Fig. 2). This analysis identified four clades: clade 1 (*n* = 58), clade 2 (*n* = 42), and clade 3 (*n* = 63), which correspond to the *K. grimontii*, *K. pasteurii*, and *K. michiganensis* phylogroups, respectively. Clade 4 included seven type strains: *K. oxytoca, K. huaxiensis, K. africana, K. indica, K. pneumoniae, K. quasipneumoniae,* and *K. variicola*. The phylogenetic analysis revealed that strains within different phylogroups were primarily grouped by sequence type rather than by their source of isolation. For example, the plant endophytic bacterium NGB-FR89 (ST127) clustered with other isolates of a similar sequence type from clinical sources in different countries.

Phylogenomic analysis also indicated that the GenBank and BV-BRC databases misclassified several strains (*n* = 7). We confirmed the new taxonomic positions of these strains using the TYGS platform. For instance, strains AACKY (GenBank: CP029770.1), SA2 (GenBank: JNFT00000000.1), and B106 (GenBank: CP067093.1) were misassigned as *K. michiganensis*, but we identified them as *K. pasteurii*. Similarly, strains CPO045 (GenBank: JARTTW000000000.2), P93 (GenBank: JBALHS000000000.1), PSU_35 (GenBank: JBCIVG000000000.1), and Survcare302 (GenBank: JAFHEI000000000.1) were misclassified as *K. grimontii* and were instead assigned to *K. pasteurii*.

**Fig. 3:**
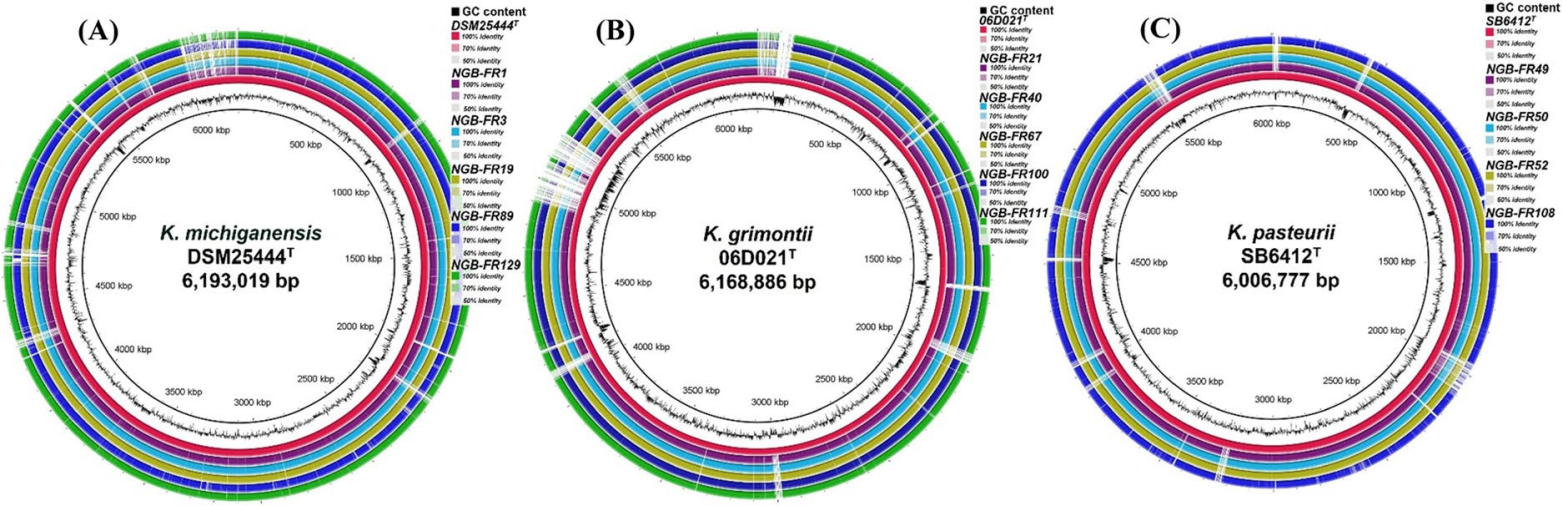
Comparative genome mapping of the 14 assembled genomes using the BLAST ring image generator BRIG v0.95. The inner cycles represented the size of closely related type strains, which were used as reference genomes as follows: **A** *K. michiganensis* DSM25444^T^, (**B**) *K. grimontii* 06D021^T^, and (**C**) *K. pasteurii* SB6412^T^*.* Each ring represents a genome, and the rings were color-coded; the genome names were labeled, and the shade of each color indicates the similarities between all strains and the reference strain

Genome comparisons among different strains within each phylogroup relative to their respective type strains revealed substantial regions of high similarity (Fig. 3). However, the genome sizes of *K. michiganensis* strains (NGB-FR1, 3, 19, 89, and 129) and the *K. grimontii* strains (NGB-FR21, 40, 67, 100, and 111) were smaller than those of their respective type strains: DSM 25444^ T^ (6.19 Mb, GenBank: PRDB01000001.1) and 06D021^T^ (6.16 Mb, GenBank: FZTC01000001.1). The genome sizes of *K. pasteurii* strains (NGB-FR49, 50, and 52) were nearly identical to that of their respective type strain SB6412^T^ (6.01 Mb, GenBank: CABGHC010000001.1), whereas strain NGB-FR 08 had a smaller genome size (5.76 Mb).

**Fig. 4:**
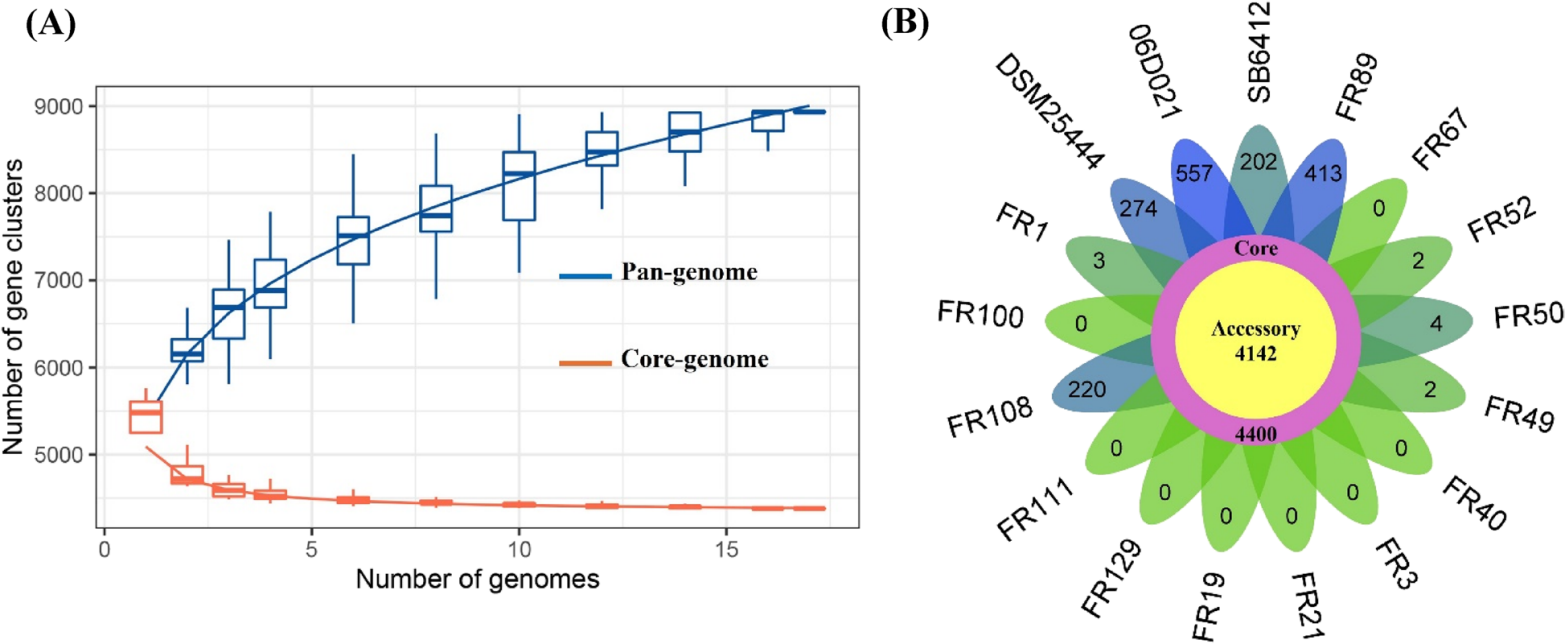
Core and pangenome analysis of the 14 strains sequenced in this study compared to their respective type strains, based on clusters of orthologous groups. **A** The number of pan-gene clusters (blue) and core gene clusters (orange) for each tested strain. **B** The flower plot illustrates the number of core and accessory genes (dispensable and strain-specific) for each strain. The center displays the total number of core and accessory genes, while the petals indicate the number of strain-specific unique genes

To identify the unique regions in the genome sequences of 14 strains compared to their relative type strains, we performed pan-genome analysis using the IPGA webtool. The results demonstrated a consistent rise in the number of pan-genomes and a decrease in the number of core-genomes with the addition of more genomes (Fig. 4a). A total of 8,542 clusters of orthologous groups (COG) were identified, consisting of 4,400 core genes common to all strains (Supplementary Table S5). Furthermore, 4,142 dispensable genes and 1,677 genes unique to different strains were detected (Fig. 4b and Supplementary Table S5). Within *K. pasteurii* genospecies, strain NGB-FR108 had the highest number of strain-specific genes (*n* = 220) compared to its relative type strain SB6412^T^ (*n* = 202). For *K. michiganensis* genospecies, strain NGB-FR89 exhibited the largest number of strain-specific genes (*n* = 413), relative to its corresponding type strain DSM25444^T^ (*n* = 274). The five strains of *K. grimontii* genospecies had no strain-specific genes, while the type strain 06D021^T^ possessed a total of 557 strain-specific genes. In general, these strain-specific genes were predominated by unannotated sequences (68%; Supplementary Fig. S2). However, the remaining genes were related to the “metabolism” COG class, followed by the “cellular processes and signaling” and the “information storage and processing” COG categories (Supplementary Fig. S3).

### Genes involved in PGP activities and abiotic stress tolerance

**Fig. 5:**
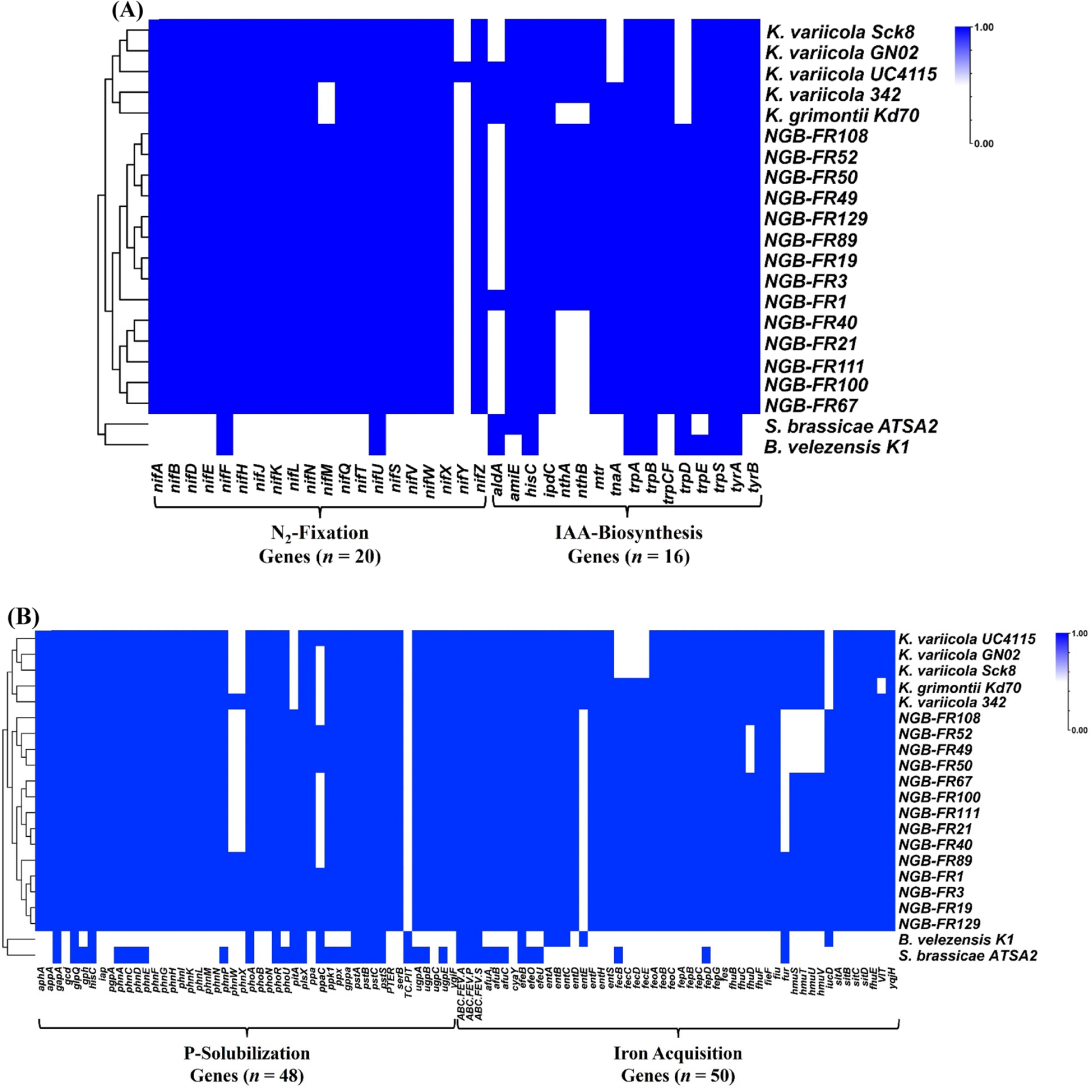
Heatmap showing the presence/absence of predicted PGP genes within the 14 assembled genomes sequenced in this study, compared to seven efficient PGP reference strains. **A** Presence/absence of PGP genes associated with N₂ fixation and IAA biosynthesis. **B** Presence/absence of PGP genes related to P-solubilization and iron acquisition traits. The blue color indicates the presence of PGP genes, while white indicates their absence. Detailed information regarding the PGP profiles is provided in Supplementary Table S6

**Fig. 6:**
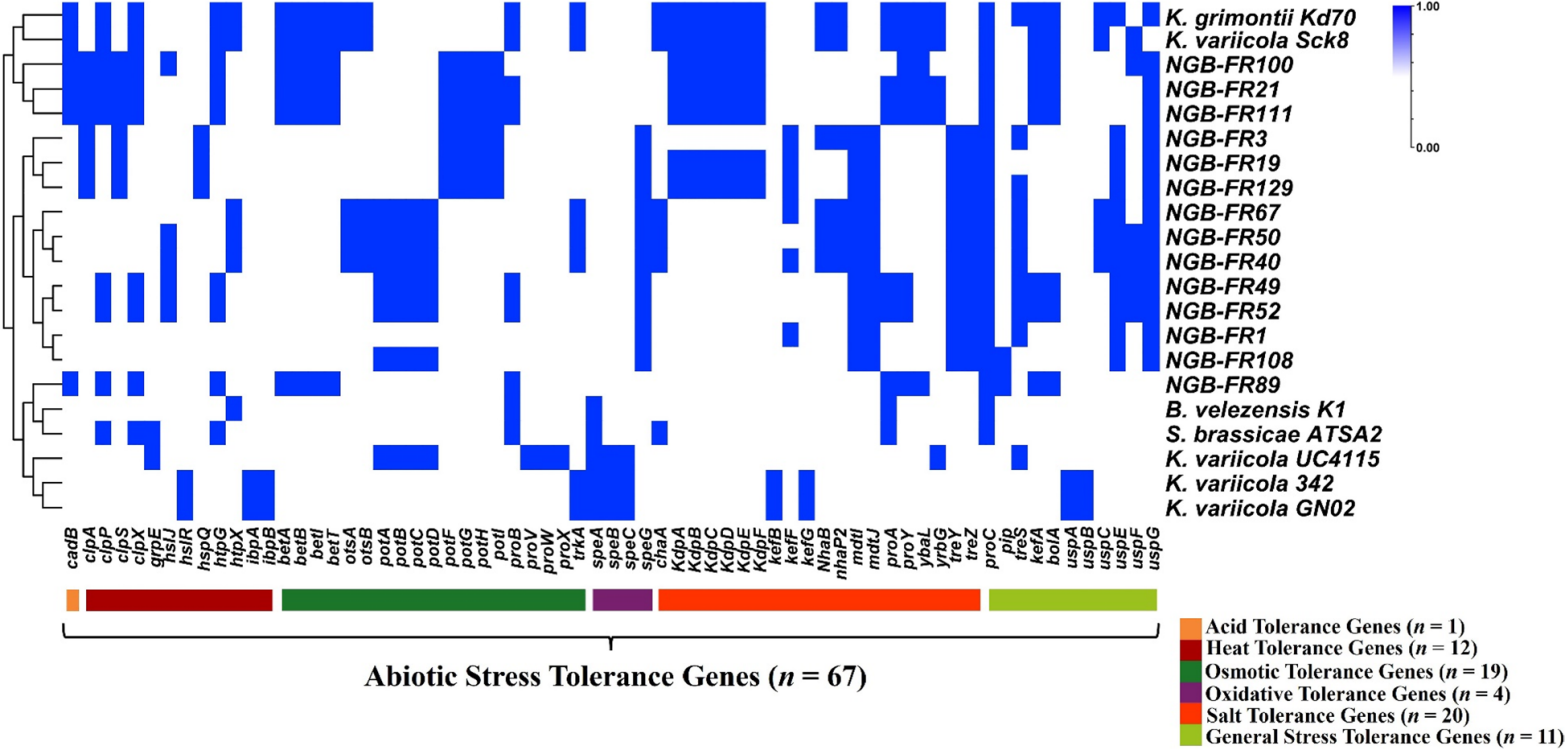
Heatmap showing the presence/absence of predicted genes associated with abiotic stress tolerance genes within the 14 assembled genomes sequenced here, compared to seven efficient PGP reference strains. The blue color indicates the presence of abiotic stress tolerance genes, while white indicates their absence. Detailed information regarding the PGP profiles is provided in Supplementary Table S6

We annotated the 14 genomes studied here to identify genes implicated in PGP-related pathways and those associated with abiotic stress tolerance. Additionally, we included the genomes of previously reported efficient PGP strains: *K. grimontii* Kd70 (an endophyte from the intestine of *Diatraea saccharalis* larvae), *K. variicola* 342 (an endophytic diazotroph from maize), *K. variicola* G02 (an endophytic diazotroph from *Pennisetum sinense* Roxb.), *K. variicola* Sck8 (a rhizospheric diazotroph from a sugarcane field), *K. variicola* UC4115 (a rhizospheric bacterium of tomato plant), *B. velezensis* K1 (an endophyte from a banyan tree), and *S. brassicae* ATSA2 (an endophyte from kimchi cabbage). The annotation of 14 genomes revealed the presence of multiple genes associated with plant-beneficial traits and abiotic stress tolerance, which were comparable to those found in other previously reported PGP *Klebsiella* strains and higher than those in *B. velezensis* K1 and *S. brassicae* ATSA2 (Figs. 5 & 6 and Supplementary Table S6).

All 14 genomes, along with all *Klebsiella* reference genomes, possessed 20 *nif* genes (*nifABDEFHJKLMNQSTUVWXYZ*) involved in the nitrogen fixation process (Fig. 5 and Supplementary Table S6). However, *B. velezensis* K1 and *S. brassicae* ATSA2 only contained the *nifF* and *nifU* genes. We found numerous genes (*n* = 16) implicated in IAA production within the draft genomes of the 14 strains (Fig. 5 and Supplementary Table S6). The *nthA-B* genes, coding for the nitrile hydratase enzyme, were exclusively identified in the genomes of *K. michiganensis* and *K. pasteurii*. This enzyme converts indole-3-acetonitrile (IAN) to indole-3-acetamide (IAM). The *amiE* gene, present in all 14 strains, encodes amidase that converts IAM into IAA. All 14 genomes also contained the *ipdC* gene, which encodes pyruvate decarboxylase, responsible for converting indole-3-pyruvic acid (IPA) to indole-3-acetaldehyde (IAD). Notably, only *K. michiganensis* NGB-FR1 had the *aldA* gene, which encodes aldehyde dehydrogenase, an enzyme that converts IAD into IAA. The *tnaA* gene encoding tryptophanase, which oxidizes tryptophan to indole, was also found in all 14 genomes. The test strains also had several genes, including histidinol phosphate aminotransferase (*hisC*), tryptophan-specific transport protein (*mtr*), and the *trp* gene cluster (*trpABCFDES*). These genes are involved in the production of tryptophan, the precursor for IAA biosynthesis.

Most of the assembled genomes contained a substantial number of genes (*n* = 48) associated with P solubilization (Fig. 5 and Supplementary Table S6). We identified seven genes that contribute to inorganic P solubilization, including glucose dehydrogenase (*gcd*), exopolyphosphatase (*ppx*), polyphosphate kinase (*ppk1*), triphosphatase (*ygiF*), and pyrophosphatase (*ppa* and *ppaC*). Notably, we found the *ppaC* gene in only six genomes among the 14 genomes. Additionally, we found 25 genes involved in organic P mineralization, particularly those related to phosphonate and phosphinate metabolism. We also detected three genes associated with P-regulation (*phoB*, *phoR*, and *phoU*) and 13 genes related to P-transportation in all the 14 genomes.

In the context of iron acquisition, we found that most of the assembled genomes contained genes (*n* = 50) involved in iron metabolism and siderophore biosynthesis, particularly the enterobactin catecholate-type siderophore (Fig. 5 and Supplementary Table S6). We identified the ferric chelate reductase (*yqjH),* a siderophore-interacting protein that is essential for the release of iron from various iron chelators*.* We also recognized the *ent* gene cluster *(entABCDEFHS*), the *feb* gene cluster (*fepABCDG*), and the iron (III)-enterobactin esterase (*fes*), which are responsible for enterobactin biosynthesis and export. It is important to note that only the 14 assembled genomes and *B. velezensis* K1 had the lysine N6-hydroxylase (*iucD*) that is associated with the biosynthesis of aerobactin (a citrate-hydroxamate siderophore). Furthermore, we found two transcriptional-regulating genes (*feoC* and *fur*) as well as 30 genes that are implicated in iron transport. Interestingly, we detected an iron-sulfur cluster assembly protein (*cyaY*) in all test strains.

The annotation results revealed that various strains in this study harbor numerous genes that enhance plant tolerance to environmental stresses (Fig. 6 and Supplementary Table S6). Among the 14 analyzed genomes, only NGB-FR 21, 89, 100, and 111 possessed the lysine:cadaverine antiporter (*CadB*), a critical component for acid tolerance and the maintenance of pH homeostasis within cells. Several strains also had the *clpAPSX* ATPases and *htpGQX* heat shock proteins, which are associated with heat stress tolerance and improved responses to elevated temperatures. In contrast, other chaperone proteins, such as *grpE*, *hslR*, and *ibpAB*, were absent in all examined strains. We found 16 genes that are linked to osmotic stress tolerance in different tested strains. These include the *bet* operon (*betABIT*), which is involved in the biosynthesis of the osmoprotectant glycine betaine, and the *pot* operon (*potABCDFGHI*), which facilitates the import of polyamines (putrescine and spermidine). Notably, only *K. grimontii* strains NGB-FR 40, 67, Kd70, and *K. variicola* Sck8 exhibited trehalose 6-phosphate synthesis genes (*ostAB*), which play a regulatory role in responses to various stressors, including osmotic stress. Most strains (71%, 10/14) exhibited diamine N-acetyltransferase (*speG*), which is associated with proline metabolism and oxidative stress tolerance. We analyzed 20 salt tolerance genes and found 18 of them in several strains. For instance, the potassium transport operon (*KdpABCDEF*), the trehalose-producing operon (*treYZ*), the spermidine exporter operon (*mdtIJ*), the proline biosynthesis operon (*proAY*), and the calcium/sodium:proton antiporter (*yrbG*) all significantly contribute to salt tolerance. Furthermore, we identified general stress response genes associated with resistance to multiple stresses, such as the *uspCEFG* genes that encode universal stress proteins (USPs).

We confirmed the abiotic stress tolerance activity of the 14 strains tested here by assessing their growth across a wide range of salt concentrations and temperatures in vitro (Supplementary Table S7). All strains were salt-tolerant and exhibited tolerance activity ranging from 3–4% NaCl. The most tolerant strains were *K. grimontii* NGB-FR21 and 111, which could grow at 4% NaCl. Regarding tolerance to high temperatures, large numbers of the tested strains (57%, 8/14) were tolerant up to 42 °C, while three *K. grimontii* strains (NGB 21, 100, and 111) could thrive at temperatures up to 45 °C.

### Antimicrobial resistance, virulence genes, and MEGs in KoSC

**Fig. 7:**
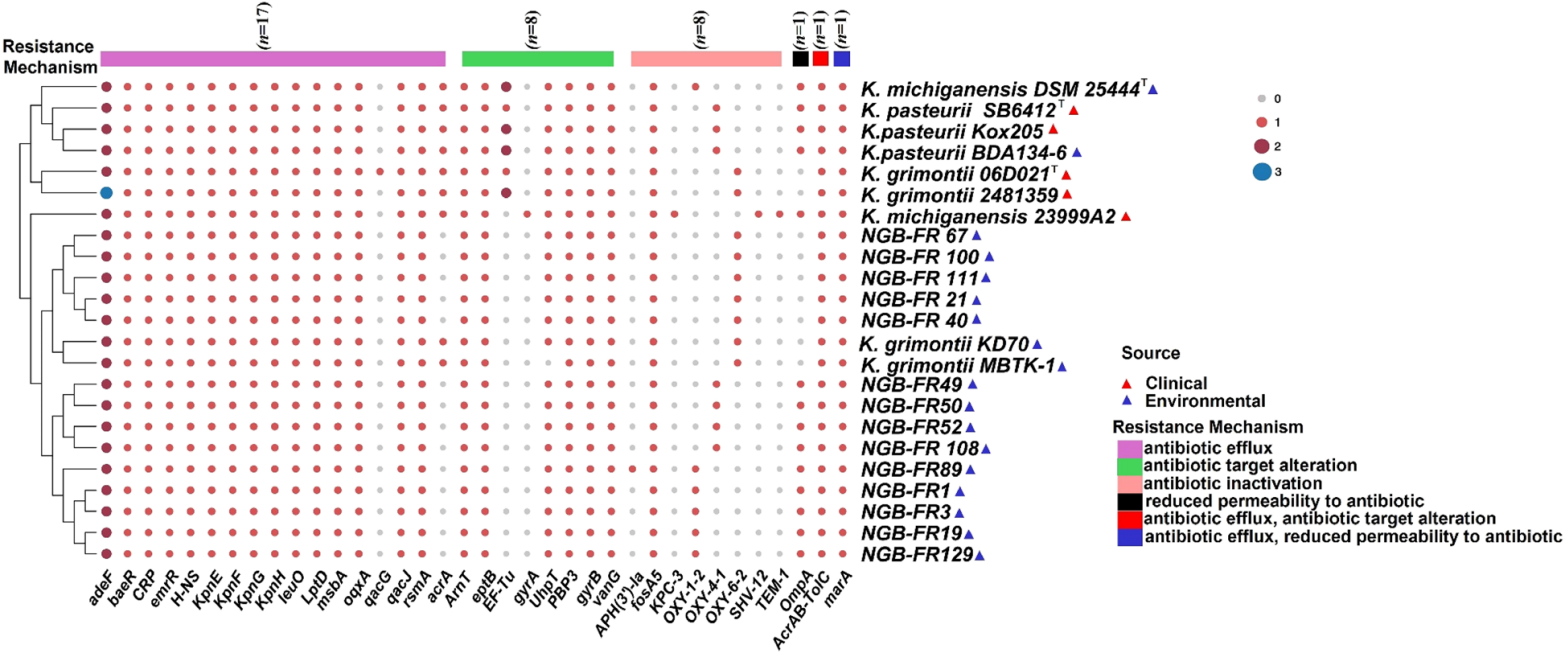
Heatmap of the antibiotic resistance genes (*n* = 36) and their relative copy numbers in the 14 assembled genomes sequenced here compared to nine reference genomes of closely related *Klebsiella* species isolated from clinical and environmental sources. The antibiotic resistance profiles of all bacterial strains were screened using the CARD database. Genes are grouped according to their resistance mechanisms

The resistance profile and the whole ARGs spectrum among the 14 strains were comparatively similar. Resistome analysis indicated the presence of 29 ARGs distributed among the assembled genomes; however, only 24 of these were prevalent across all genomes, conferring resistance to a range of antibiotics (Fig. 7 and Supplementary Table S8). These genes are responsible for three main resistance mechanisms: antibiotic efflux, antibiotic target alteration, and antibiotic inactivation. The predominant resistance mechanism identified was antibiotic efflux, mediated by 15 genes, which conferred resistance to multiple classes of antibiotics, including fluoroquinolone, macrolide, cephalosporin, aminoglycoside, and tetracycline. An additional resistance mechanism named reduced permeability to antibiotics, mediated by the *OmpA* gene, was exclusively identified in strains of *K. michiganensis* and *K. pasteurii*, accounting for 64% of the tested strains. Interestingly, we identified two genes involved in multiple resistance mechanisms. The first is the *AcrAB-TolC* gene responsible for antibiotic efflux and target alteration, while the second is the *marA* gene involved in antibiotic efflux and reduced permeability. All 14 genomes harbored the chromosomally encoded beta-lactamase gene (*bla*_*OXY*_), which is intrinsic to the *K. oxytoca* complex. Each species carried distinct variants of the *bla*_*OXY*_ gene: *K. michiganensis* carried *bla*_*OXY-1–2*_, *K. pasteurii* contained *bla*_*OXY-4–1*_, and *K. grimontii* possessed *bla*_*OXY-6–2*_. Remarkably, all 14 genomes lacked the previously recognized extended-spectrum beta-lactamase (ESBL) encoding genes, such as *bla*_*TEM-1*_ and *bla*_*SHV*_, nor the carbapenemase genes, such as *bla*_*KPC*_, which were detected only in the genome of the clinical strain *K. michiganensis* 23999A2.

**Fig. 8:**
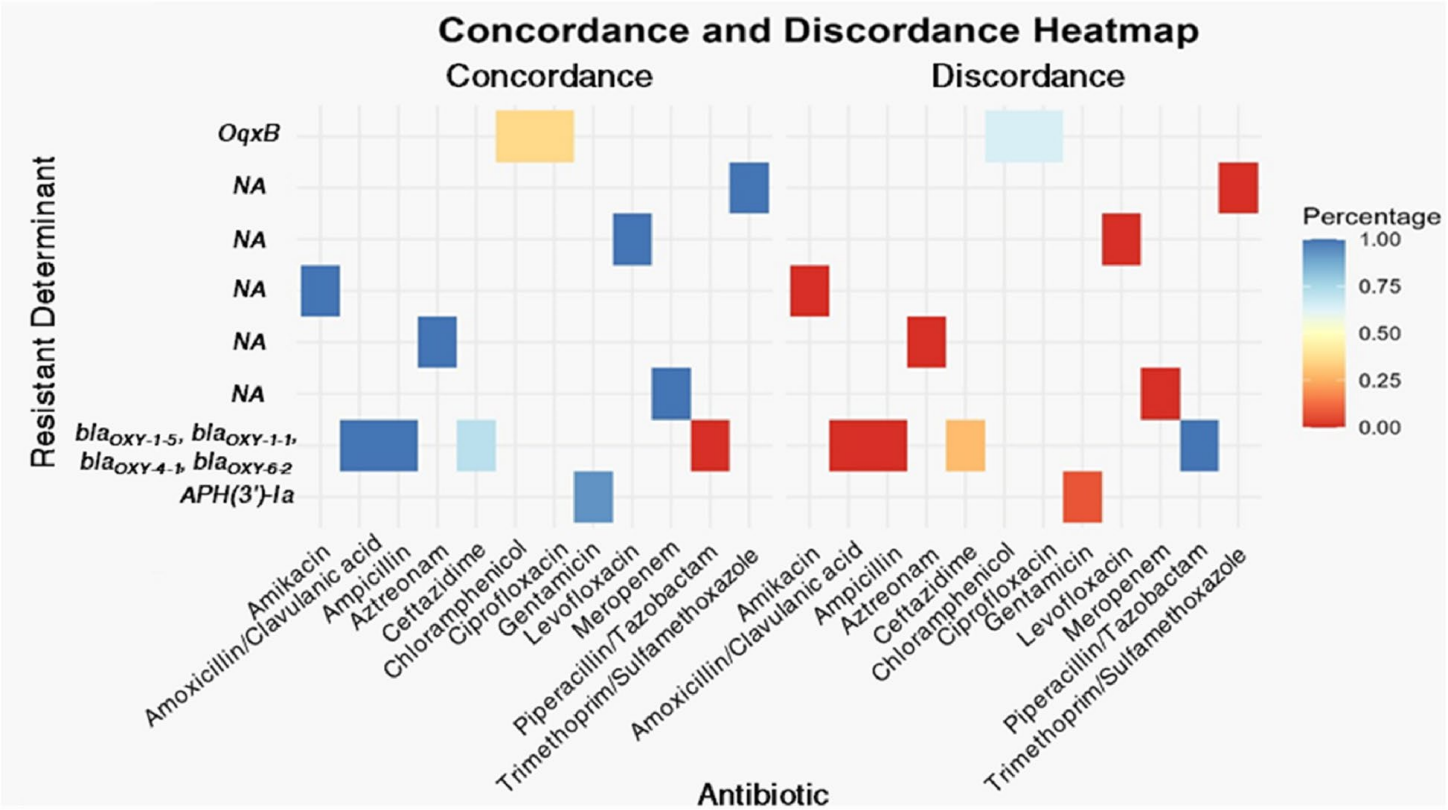
Heatmap displays the concordance and discordance between AMR genes and phenotypic susceptibility profiles across various antibiotics. The left side of the heatmap indicates concordance, where the presence of an AMR gene aligns with observed resistance, while the right-side highlights discordance, where the presence of an AMR gene does not match phenotypic resistance

We identified a strong correlation between AMR phenotypes and the prediction of ARGs in the assembled genomes (Fig. 8). Full concordance (100% of isolates) was observed for several antibiotics, including ampicillin, meropenem, amoxicillin/clavulanic acid, aztreonam, amikacin, levofloxacin, and trimethoprim/sulfamethoxazole. In contrast, significant discordance was noted between in vitro antibiotic susceptibility and genetic determinants for gentamicin (7%), ceftazidime (29%), ciprofloxacin (64%), chloramphenicol (64%), and piperacillin/tazobactam (100%).

**Fig. 9:**
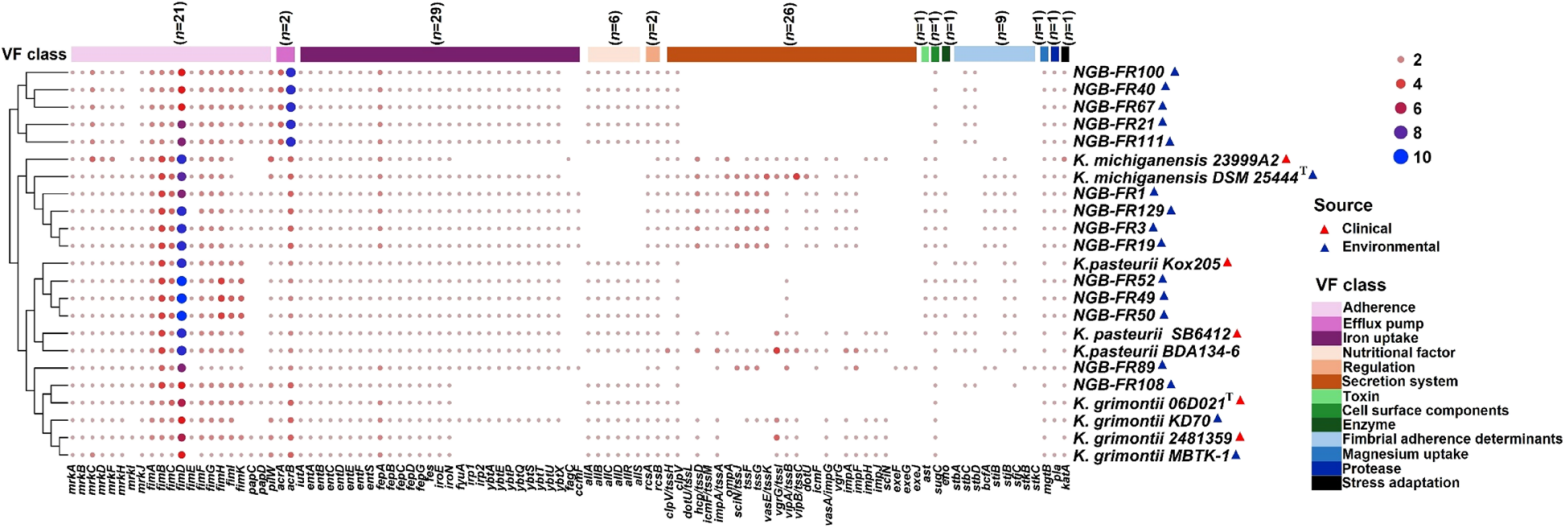
Heatmap of the virulence factors (*n* = 101) and their relative copy numbers in the 14 assembled genomes sequenced here compared to nine reference genomes of closely related *Klebsiella* species isolated from clinical and environmental sources. The virulome profiles of all bacterial strains were screened using the VFDB database. Genes are grouped according to their virulence classes

Analysis using the VFDB revealed a diverse distribution of 95 virulence-related genes among our strains (Fig. 9 and Supplementary Table S9). These genes predominantly fell into three key categories: adherence (*n* = 21), iron uptake (*n* = 29), and secretion system (*n* = 20). Approximately 50% (*n* = 47) of the identified virulence genes were present across all assembled genomes. Most tested strains possessed the *fim* and *mrk* operons, which encode type 1 and type 3 fimbriae responsible for adherence and biofilm formation. In addition, many genomes had acquired virulence-encoding genes (*stbACD*, *bcfA*, *stiB*, *stjBC*, and *stkBC*) that are responsible for the synthesis of other fimbrial adherence factors, including fimbrial chaperone proteins. The virulome analysis revealed that the strains of *K. grimontii* and *K. pasteurii* only had the *stbCD* genes, while strain *K. michiganensis* NGB-FR89 only had the *stkBC* genes. All assembled genomes carried genes encoding the iron-chelating siderophores aerobactin, enterobactin, and salmochelin. Moreover, all strains of the *K. michiganensis* and *K. grimontii* groups harbored yersiniabactin-encoding genes, whereas only 75% of strains in the *K. pasteurii* group contained these genes. Genes associated with the Type VI Secretion System (T6SS), specifically T6SS-I, T6SS-II, and T6SS-III, were more prevalent in *K. michiganensis* strains compared to those in the *K. grimontii* and *K. pasteurii* groups. Of note, the *exe*-acquired gene cluster (*exeFGJ*), which encodes the type II secretion system (T2SS), was only present in the *K. michiganensis* NGB-FR89 genome.

The PlasmidFinder tool identified three plasmid replicons that do not encode any antibiotic resistance genes (Supplementary Table S10). The IncF family plasmids (FII and/or FIB replicons) were detected in eight strains of *K. michiganensis* (NGB-FR1, FR3, FR19, FR89, and FR129) and *K. pasteurii* (NGB-FR49, FR50, and FR52). The most frequently detected plasmid replicons were IncFII(Yp) and IncFIB(K), which were identified in seven strains. In contrast, the IncFIB (pKPHS1) replicon was found only in the *K. michiganensis* NGB-FR 89 strain. Additionally, the Col440I plasmid replicon was identified exclusively in strains of *K. michiganensis*. The IncFIB and Col440I plasmid replicon types correspond to identifications associated with *K. pneumoniae*. However, the IncFII plasmid replicon exhibited 95.2% similarity to the plasmid MT of *Yersinia pestis* (Accession No. CP000670.1).

Furthermore, the MobileElementFinder tool identified several insertion sequences related to the IS3 family transposase (*ISEcl10*, *ISEc15*, *ISEch12*, and *ISEam1*) and the IS110 family transposase (*ISCfr13* and *ISKpn2*) (Supplementary Table S11). Further investigation of MGEs using the mobileOG-db database revealed numerous sequences encoding various proteins that mediate essential features of bacterial MGEs (Supplementary Fig. S4 and Table S12). These MGEs were categorized into five groups based on their functions. The category encompassing replication, recombination, and repair contained the largest number of sequences, ranging from 117 to 146. To elucidate the role of MGEs in the dissemination of antibiotic resistance, we analyzed the ARGs associated with these MGEs (Supplementary Table S13). Only three ARGs were commonly linked to the identified MGEs: *ompA*, which is associated with transfer function and confers resistance to peptide antibiotics; *gyrB*, involved in replication, recombination, and repair, which provides resistance to fluoroquinolone antibiotics; and *H-NS*, also related to replication, recombination, and repair, which confers resistance to fluoroquinolones, cephalosporins, penicillin beta-lactams, and tetracyclines.

## Discussion

The utilization of PGP bacteria as biostimulants for cultivated crops constitutes a promising technological advancement, demonstrating superior efficacy and environmental sustainability relative to chemical fertilizers [12]. In legumes, root nodules not only host symbiotic rhizobia but also contain significant populations of non-symbiotic endophytes that play a pivotal role in the nodulation process and contribute to overall plant growth and fitness [18]. These nodule-endophytes are likely to exhibit beneficial effects and mechanisms analogous to those of PGP bacteria, proving potential as biofertilizers and biocontrol agents [35]. The most identified endophytic bacteria in the root nodules include *Bacillus* and *Pseudomonas*, followed by *Paenibacillus*, *Agrobacterium*, and *Enterobacter* [35]. However, in a previous study, we isolated and characterized 14 endophytic strains belonging to the KoSC from the root nodules of faba bean plants [18]. These strains improved nodulation and had multiple PGP attributes such as P-solubilization and siderophore production [18]. In the present study, WGS was done to elucidate the genomic basis of the PGP traits shown by these strains. Notably, several strains from the genus *Klebsiella* have been previously identified in root nodules of numerous legumes [35, 36]. Studies have shown that these *Klebsiella* strains significantly enhanced the growth of their hosts under greenhouse and field conditions [36]. Nevertheless, the molecular mechanisms for growth promotion still need to be further explored. To the best of our knowledge, this is the first report on the genomic analysis of KoSC members isolated from the root nodules of leguminous plants.

Previous research showed that the number and arrangement of *nif* genes and *nif*-related genes associated with nitrogen fixation varied among diazotroph species, influenced by their environmental habitats [37]. However, an essential minimal set of *nif* genes (*nifHDKBEN)* was proposed for the active nitrogen fixation process [37]. For decades, the *nif* gene cluster in *Klebsiella* species*,* consisting of 20 genes arranged into eight operons, has served as a model system for investigating the genetic framework of biological nitrogen fixation [38]. The WGS demonstrated that all strains sequenced in this study contained the complete *nif* operon (20 genes) responsible for nitrogen fixation. Comparable findings were found in the genomes of previously reported endophytic nitrogen-fixing strains belonging to the KoSC [10, 39].

IAA, the most common plant auxin, is generally synthesized via the L-tryptophan (Trp) metabolism pathway or via the Trp-independent pathway [40]. In bacteria, four major Trp-dependent pathways for IAA synthesis have been identified: IPA, IAM tryptamine (TPM), and indole-3-acetaldoxime/indole-3-acetonitrile (IAOx-IAN) pathways. Consistent with earlier studies demonstrating that multiple synthesis pathways can coexist within a single bacterium [41], WGS results indicated that most strains sequenced here had several genes associated with different IAA biosynthesis pathways. Here, two main pathways, the IAM and IPA pathways, were proposed in the genomes of the 14 tested strains. This was evidenced by the presence of the *amiE* gene, which contributes to the conversion of IAM into IAA, and the *ipdC* gene, which is responsible for the IPA decarboxylation. The IAM and IPA pathways have been commonly reported in PGP bacteria [40]. In addition, strains of *K. michiganensis* and *K. pasteurii* harbored *nthA*-*B* genes involved in the IAOx-IAN pathway, which contributed to the conversion of IAN to IAM and finally to IAA. Notably, only *K. michiganensis* NGB-FR1 contained the *aldA* gene, which metabolizes indole-3-acetamide (IAD), a critical intermediate in both the IPA and TPM pathways, into IAA. Remarkably, all genomes analyzed included the *trpCF*, which encodes indole-3-glycerol phosphate synthase, a putative key enzyme in the Trp-independent pathway [42]. While previous studies reported the presence of Trp-independent pathways in bacteria [43], further investigation is necessary, as the key enzymes and genes involved in this pathway are still unconfirmed. In agreement with our results, the high potential for IAA production among tested strains, particularly *K. michiganensis* [10, 44]. For instance, consistent with our findings, *K. michiganensis* LDS17, isolated from the rhizosphere of *Codonopsis pilosula*, exhibited four genes associated with two IAA synthetic pathways: *nthA-B* genes contributed to the IAOx-IAN pathway, and indolepyruvate decarboxylase and amidase genes related to the IPA pathway [9].

PGP microbes with phosphate-solubilizing activity play a vital role in enhancing P-bioavailability, thereby facilitating its absorption by plants and ultimately increasing crop yields [45]. In this study, we propose that our strains utilize two primary mechanisms for phosphate solubilization: the secretion of organic acids and the production of enzymes that dissolve recalcitrant soil phosphorus. We identified genes associated with acidolysis in the 14 genomes, including *gcd*, which encodes glucose dehydrogenase, and *gapA*, which encodes glyceraldehyde 3-phosphate dehydrogenase. In line with our data, previous research has reported that PGP *Klebsiella* spp. enhanced P-bioavailability by secreting various organic acids [46]. Additionally, we found numerous genes encoding phosphate-solubilizing enzymes that have been extensively studied in prior research [47], such as alkaline phosphatase (*phoA*), acid phosphatases (*aphA*, *appA*, and *phoN*), phosphoglycolate phosphatase (*gph*), inorganic pyrophosphatase (*ppa*), extracellular polyphosphatase (*ppx*), and polyphosphate kinase (*ppk1*). Furthermore, we identified several genes involved in regulating the phosphorus deprivation response (*phoB*, *phoR*, and *phoU*), as well as those related to phosphorus uptake and transport (*pst*, *ugp*, and *phn* operons), which are particularly activated under conditions of limited phosphorus availability [48]. These findings corroborate our previous data, demonstrating the effective capability of these strains to solubilize inorganic phosphate with a range of 55–136 µg mL^−1^ [18].

Iron is an essential element necessary for various biological functions; however, its acquisition poses significant challenges for many microorganisms [49]. While mechanisms by which bacteria uptake ferric iron have been extensively investigated, the mechanism for ferrous iron uptake remains less clearly defined [50]. Notably, a functional overlap for iron acquisition has been identified across all assembled genomes. For example, the *EfeUOB* system, which facilitates the transport of both ferric and ferrous iron, was observed in all genomes studied. Siderophores are small iron-binding molecules that enable bacteria to sequester iron from their environment and transport it into their cells [49]. Siderophore-producing bacteria not only promote plant growth but also reduce the impact of soil plant pathogens [45]. Most of the siderophore-related protein families identified in the sequenced genomes belonged to the biosynthesis and transport of the three types of siderophores: catecholates, carboxylates, and hydroxamates [49]. Specifically, the *ent* and *fep* operons, which are involved in the biosynthesis and transport of the catecholate siderophore enterobactin, as well as the *fhu* operon responsible for transporting ferric hydroxamate-type siderophores and the *fec* operon essential for the uptake of ferric citrate (the predominant carboxylate siderophore), were present in all genomes analyzed. Several types of siderophores have been characterized in different *Klebsiella* species, contributing to their growth efficiency and ability to colonize diverse tissues [51, 52]. Additionally, heme serves as a vital iron source for bacteria, which can acquire it through the expression of specific membrane receptors and transport proteins, such as the *hmu* operon, found in all assembled genomes. Furthermore, orthologs of the ferric uptake regulator (*fur*) protein, which plays a key role in regulating iron homeostasis, and the *feo* operon, which regulates the transport of ferrous iron, were also present in the sequenced genomes. The functional overlap in iron acquisition observed in these genomes has been previously reported in the genomes of effective plant growth-promoting bacteria [53].

In addition to improving nutrient uptake, PGP bacteria provide significant advantages in tolerating abiotic stress factors, thereby enhancing plant resilience [12]. In this study, we identified orthologs involved in the biosynthesis, binding, and transport of various osmoprotectants, including choline, betaine, trehalose-6-phosphate, glycine, and spermidine/putrescine, within the assembled genomes. These compatible osmolytes play a crucial role in mitigating osmotic, drought, and oxidative stresses [54]. Additionally, several genes encoding heat shock chaperones and universal stress proteins were observed in the sequenced genomes. Among the 14 genomes analyzed, four are equipped with potassium (*NhaP2*) and sodium (*NhaB*) antiporter systems, which aid these bacteria in regulating ionic imbalances under stressful conditions [54, 55]. Notably, we found a correlation between the performance and growth of bacterial strains under abiotic stress and the genetic determinants present in their genomes. The results of our study aligned with a previous study reported by [56], who found numerous genes involved in stress tolerance mechanisms, including those related to the biosynthesis of spermidine and trehalose, in the genome of *Klebsiella* sp. LTGPAF-6F, a drought resistance-promoting endophyte.

ARGs have been extensively identified and studied in clinical settings as well as in livestock farms, soil, and aquatic environments [57]. Nevertheless, the prevalence of ARGs in plant microbiomes has not received adequate attention [15]. Earlier research has demonstrated that ARGs present in soil can be transferred to plant tissues, ultimately posing a risk to human health as they can propagate through the food chain [58]. Little information is available regarding the ARGs and virulence factors associated with KoSC [59]*.* However, acquired antimicrobial resistance is becoming an emerging concern for this species complex [60]. Most strains (71%, 10/14) were classified as MDR, exhibiting non-susceptibility to at least one antimicrobial agent across three or more classes [61]. These strains demonstrated resistance to ampicillin, ceftazidime, and amoxicillin/clavulanic acid. However, all strains were susceptible to numerous antibiotics such as meropenem, gentamicin, ciprofloxacin, and levofloxacin. Regarding ARGs, several classes (*n* = 29) were identified within the assembled genomes, conferring resistance to a variety of antibiotics. Of note, ESBL genes such as *bla*_*KPC*,_ *bla*_*SHV*_, and *bla*_*TEM*_ present only in the MDR *K. michiganensis* 23999A2 (GenBank: JANFNZ000000000.1), were detected in our genomes. Consistent with our findings, 19 *bla*_*OXA*-*48*_-producing *K. oxytoca* strains, which did not express an ESBL, were susceptible to ceftazidime, ciprofloxacin, gentamicin, levofloxacin, and amikacin [62].

A general concordance was observed between antibiotic resistance phenotypes and their corresponding genetic determinants (predicted ARGs); however, notable discordances were evident in certain instances. For example, the presence of the *bla*_*OXY*_ gene, which encodes the chromosomal class A OXY *β*-lactamase, was not corroborated by in vitro susceptibility test results for piperacillin/tazobactam and ceftazidime, with 100% and 29% of strains, respectively, demonstrating sensitivity to these antibiotics. Similarly, 64% of the tested strains were susceptible to ciprofloxacin and chloramphenicol, despite harboring the *OqxB* resistance gene in their assembled genomes. The variability in the accuracy of genomic data for predicting AMR phenotypes within *Enterobacterales* has been frequently reported [63, 64]. Antimicrobial phenotype-genotype variation includes the presence of phenotypically susceptible isolates that carry genetic AMR determinants, as well as phenotypically resistant strains lacking known genetic resistance mechanisms [63]. Several factors may contribute to the imperfect correspondence between the presence or absence of an AMR gene and in vitro susceptibility results. These factors include differences in sequencing platforms and pipelines, incomplete comparative databases, inadequate genotyping algorithms, variability in gene expression, the presence of single-nucleotide polymorphisms (SNPs), and highly diverse polygenic backgrounds [63, 64].

The virulence profiles of the assembled genomes were examined to assess their likelihood of causing severe infections. All genomes contained a core set of pathogenicity factors (*n* = 41), coding for type I and type III fimbriae, as well as type IV pili, which are essential for adherence and biofilm formation [65]. Furthermore, 54 additional virulence factors were identified in more than half of the population, the majority of which encoded components of the T6SS-I, II, and III secretion systems. These systems play significant roles in bacterial competition, cell invasion, and in vivo colonization [66]. A variety of acquired virulence factors were also detected, including the T2SS secretion system (*exeFGJ*) and fimbrial adherence determinants (*stbACD, bcfA, stiB, stjBC,* and *stkBC*), both of which are involved in host colonization [67]. Initial analyses of the T6SS system in phytobacteria sought to elucidate its role in virulence. However, subsequent studies have suggested that these secretion systems may also confer competitive and colonization advantages to mutualistic endophytic bacteria in their interactions with plant partners, indicating that their functions are not limited to virulence alone [68]. Notably, it has been reported that the T6SS significantly enhances the capacity of PGP bacteria to colonize plant tissues [69] and improves their competitive fitness and protection against bacterivores [70]. Recent studies have further revealed that the T6SS and T2SS systems play a stimulating role in the early stages of symbiotic interaction between *Rhizobium* and legumes [67, 71]. In this context, we propose that the abundance of genes encoding secretion system proteins in the assembled genomes of our strains supports their endophytic lifestyle and enhances their colonization capacity within faba bean root nodules. Nonetheless, this advantage may pose significant risks, as these bacteria can colonize a diverse array of hosts, including humans, rather than being limited solely to plants.

At the time of writing this paper, the PubMLST database (www.pubmlst.org) contained collections of 107, 240, and 49 different STs for *K. grimontii*, *K. michiganensis*, and *K. pasteurii*, respectively. The analysis of our strains generated five sequence types: ST-576 for *K. grimontii*, ST-27 and ST-542 for *K. michiganensis*, and ST-569 and ST-629 for *K. pasteurii*. The ST-576 was exclusively identified in the *K. grimontii* 700,466–17 obtained from a urine sample in Switzerland [72]. The ST-27 is well established in *K. michiganensis* and has been identified among clinical strains in several countries, including China (*n* = 8), Switzerland (*n* = 4), Australia (*n* = 2), the USA (*n* = 2), the UK (*n* = 1), Germany (*n* = 1), and Lebanon (*n* = 1). In contrast, ST-542, ST-569, and ST-629 were novel to this study and were registered at the PubMLST database. Our findings align with previous studies on the KoSC, which have demonstrated a close genetic relationship between environmental and clinical isolates [2]. This suggests the possibility of an environmental source contributing to infections.

Due to their genomic plasticity, KoSC strains are adept at acquiring plasmids and MGEs, thereby enhancing their resistance determinants and adaptability for survival [73]. Various plasmids, belonging to different replicon types, have been previously documented within the *K. oxytoca* complex [74]. In this study, we identified conjugative plasmid replicons (IncFII and IncFIB) and a mobilizable plasmid replicon (Col440I), which are commonly linked to the spread of ARGs in *K. pneumoniae* and other members of the KoSC [74, 75]. However, the MobileElementFinder tool revealed that the plasmid replicons identified in the current investigation did not carry any antibiotic resistance genes. Similar findings were reported by [76], who noted the presence of IncF and IncR type plasmids lacking ARGs in 170 genomes of *K. variicola* isolated from a wastewater treatment plant. Additionally, we identified numerous insertion sequences belonging to the IS3 and IS110 families, which have previously been recognized as important elements for the horizontal transmission of ARGs [73]. Numerous sequences associated with MGEs, particularly those exhibiting phage and plasmid features, were predicted using the mobileOG-db database. However, analysis with the CARD indicated that only three ARGs were associated with these MGEs. This finding, however, warrants further investigation, as the incomplete recovery of plasmid sequences using short-read sequencing technologies has hindered the accurate identification of ARGs localized on specific plasmids [75, 77].

Recent studies indicated that effective regulation of biofertilizers is essential for ensuring compliance with safety standards and public health [78, 79]. To mitigate the risks associated with the rising prevalence of antibiotic resistance, stakeholders must adopt practical strategies. These strategies should include the development of comprehensive risk assessment frameworks for evaluating biofertilizer recommendations and conducting rigorous pre-application risk studies to examine the potential dissemination of ARGs [15]. Additionally, prioritizing the use of antibiotic-sensitive PGP bacterial strains while excluding multidrug-resistant variants is critical [80]. Minimizing reliance on genetically engineered strains that contain ARG biomarkers [81] and exercising caution with bacterial species closely related to pathogenic species are also advisable [78]. Employing genome mining techniques for precise characterization of PGP strains can aid in identifying biofertilizers with low potential risks and high efficacy [82]. Lastly, genomic surveillance of the antibiotic resistome in farming systems that use biofertilizers will offer valuable insights into resistance patterns and plan better ways to manage risks in the future.

## Conclusion

This study elucidates the intricate interplay between the PGP capabilities of the strains belonging to KoSC and their associated health risks stemming from antibiotic resistance. The genomic analysis of the tested strains identified significant beneficial traits that could enhance agricultural productivity. However, the detection of antibiotic resistance genes raises serious concerns regarding their implications for human health and ecosystem integrity. Our findings emphasize the potential health risks posed by plant-associated KoSC strains, as indicated by considerable MDR, the presence of novel sequence types, and the widespread occurrence of virulence and resistance genes in their assembled genomes. To our knowledge, this represents the first report offering genomic insights into the potential risks associated with legume nodule-associated bacteria, particularly those related to the KoSC. Overall, this study advocates for the establishment of a regulatory framework for the selection of bacterial-based biofertilizers, aimed at maximizing their benefits in crop improvement while minimizing the risk of spreading antibiotic-resistant bacteria.

## Supplementary Information

Supplementary Material 1.

Supplementary Material 2.

## Data Availability

The whole genome sequence of the 14 strains sequenced in the current study has been submitted to the NCBI GenBank database (https://www.ncbi.nlm.nih.gov/) in BioProject No. PRJNA923792.
