# Supplementary material for: Nodules-associated Klebsiella oxytoca complex: genomic insights into plant growth promotion and health risk assessment
Source: BMC Microbiol. 2025 May 15;25:294. doi: 10.1186/s12866-025-04002-7 (PMC12079993; doi:10.1186/s12866-025-04002-7)
Supplement: Supplementary file 1 — Supplementary Material 1. [file 12866_2025_4002_MOESM1_ESM.docx]

**Supplementary Figures**


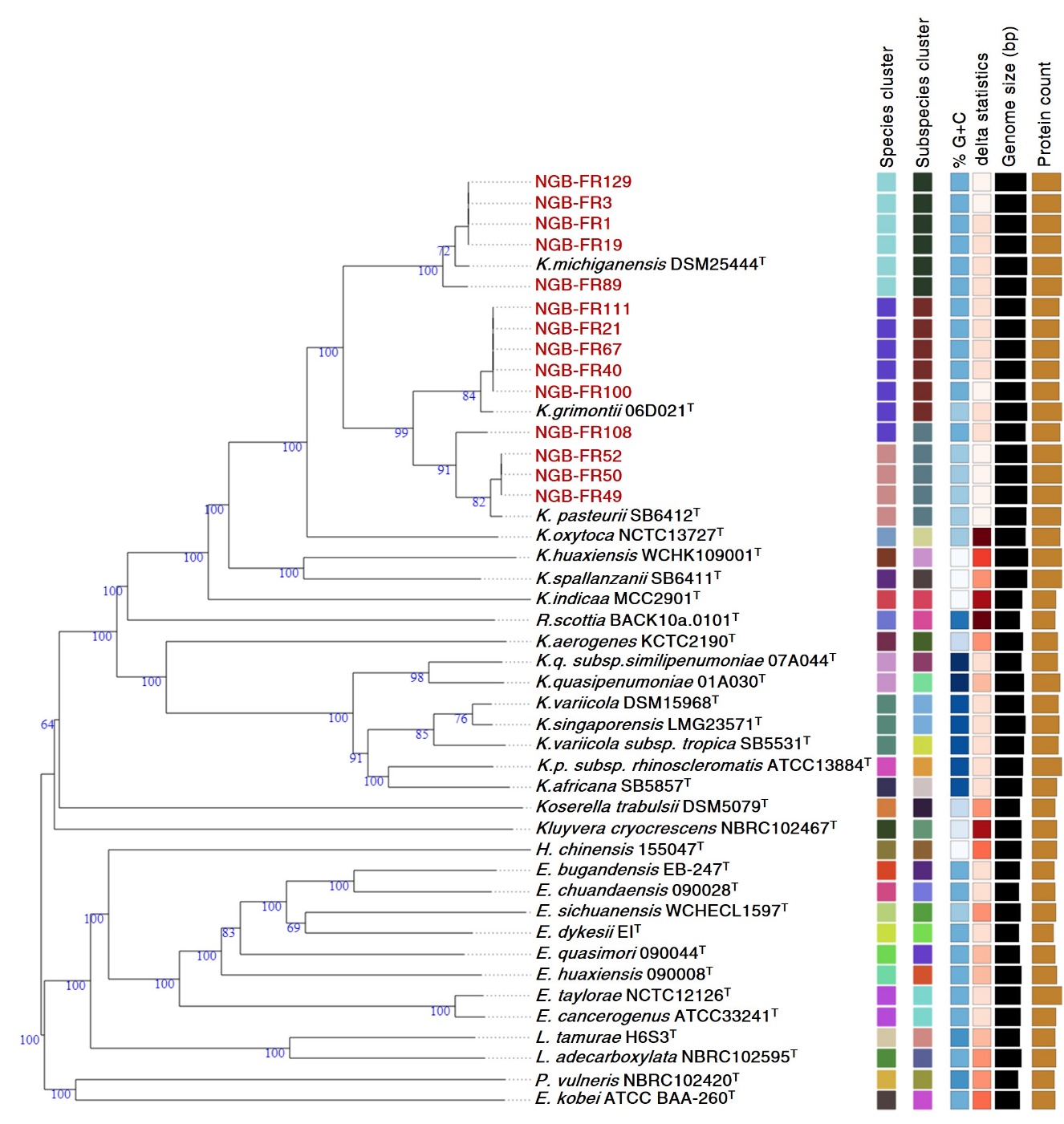


**Fig. S1** Phylogenomic relationship between the 14 strains sequenced in this study (in red) and closely related *Enterobacterial* species (*n* = 31) constructed by TYGS. Tree inferred with FastME 2.1.6.1 (Lefort et al., 2015) from GBDP distances calculated from genome sequences. The numbers above the branches represent GBDP pseudo-bootstrap support values > 60% from 100 replications, with an average branch support of 81.7%. The tree was rooted at the midpoint. *E: Enterobacter, H: Huaxiibacter, K: Klebsiella, L: Leclercia,* and *P: Pseudescherichia.*


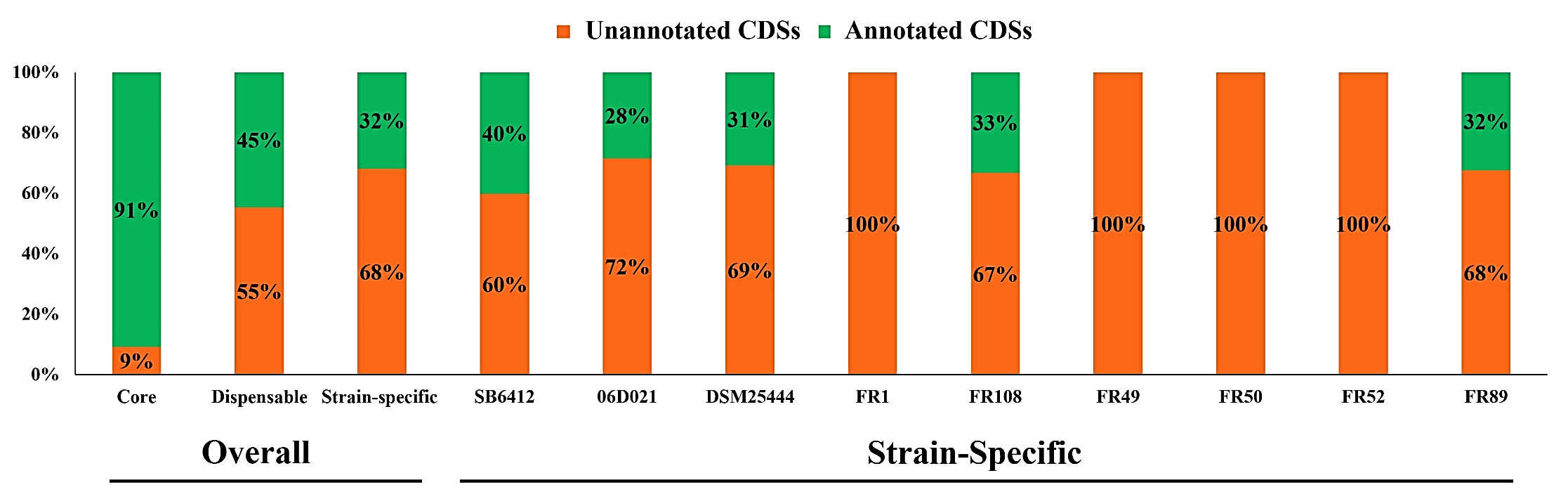


**Fig. S2** Percentage of annotated and unannotated protein-coding sequences (CDSs) in cores, dispensable, strain-specific genes in the group, and each strain according to the KEGG and COG databases using the IPGA webtool.


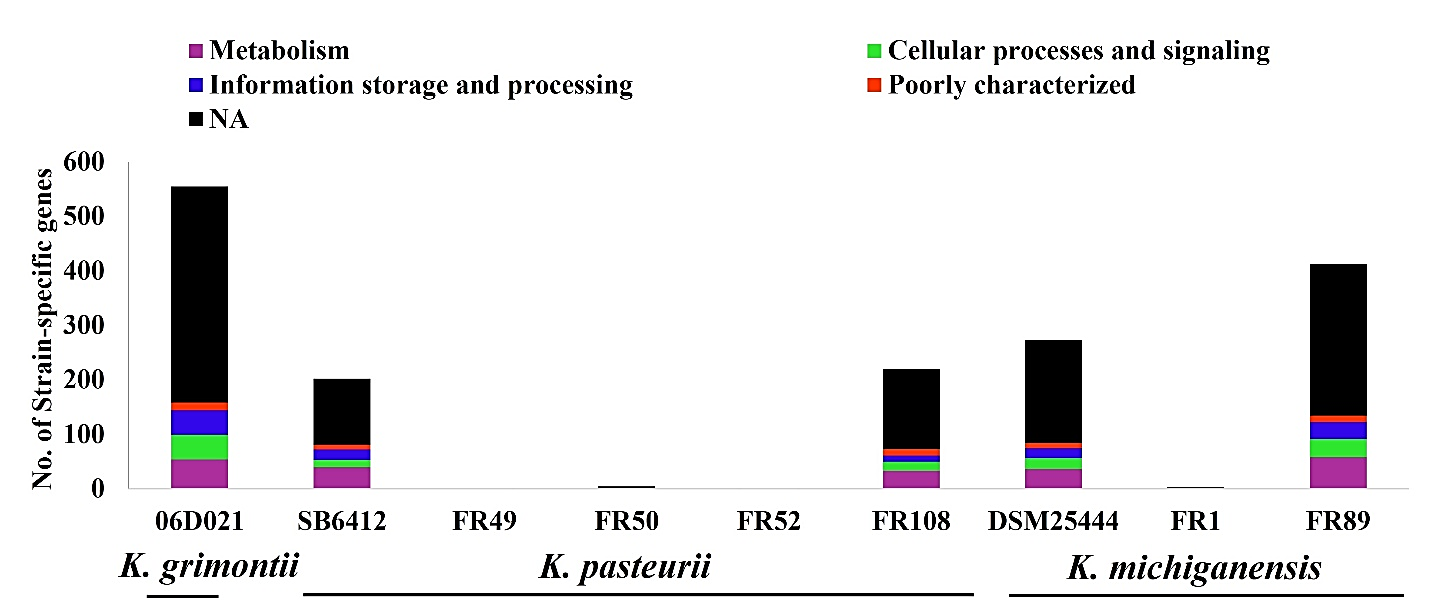


**Fig. S3** Percentages of different COG categories in strain-specific genes in each strain within the *K*. *grimontii, K. pasteurii,* and *K. michiganensis* genospecies.

**Fig. S4** List of mobile genetic elements (MGEs) identified in 14 genomes sequenced in this study, categorized and annotated using the mobileOG-db database available in the Proksee webtool
